# Gas Sensors Based on Semiconducting Nanowire Field-Effect Transistors

**DOI:** 10.3390/s140917406

**Published:** 2014-09-17

**Authors:** Ping Feng, Feng Shao, Yi Shi, Qing Wan

**Affiliations:** Jiangsu Provincial Key Laboratory of Photonic and Electronic Materials, School of Electronic Science & Engineering, Nanjing University, Nanjing 210093, China; E-Mails: fengpung@126.com (P.F.); fengangelo@hotmail.com (F.S.); yshi@nju.edu.cn (Y.S.)

**Keywords:** gas sensors, semiconducting nanowires, field effect transistors

## Abstract

One-dimensional semiconductor nanostructures are unique sensing materials for the fabrication of gas sensors. In this article, gas sensors based on semiconducting nanowire field-effect transistors (FETs) are comprehensively reviewed. Individual nanowires or nanowire network films are usually used as the active detecting channels. In these sensors, a third electrode, which serves as the gate, is used to tune the carrier concentration of the nanowires to realize better sensing performance, including sensitivity, selectivity and response time, *etc.* The FET parameters can be modulated by the presence of the target gases and their change relate closely to the type and concentration of the gas molecules. In addition, extra controls such as metal decoration, local heating and light irradiation can be combined with the gate electrode to tune the nanowire channel and realize more effective gas sensing. With the help of micro-fabrication techniques, these sensors can be integrated into smart systems. Finally, some challenges for the future investigation and application of nanowire field-effect gas sensors are discussed.

## 1. Introduction

Gas sensors are important components in safety control systems, because there are many gases that are harmful to lives and very dangerous in some circumstances [1–7]. For example, in our ordinary life, alcohol sensors are used to detect drivers' condition to reduce traffic accidents caused by drunk driving. In the medical field, monitoring a patient's respiratory system requires CO_2_ and O_2_ sensors [8]. Other typical application of gas sensors includes monitoring of toxic and flammable gas leakage in industry and civilian establishments, control of combustion efficiency and exhaust gases in automobiles [9,10]. Moreover, an unexplored future with potential applications in intelligent buildings, clean energy vehicles, or as part of advanced wireless sensor networks has been identified by scientists [11,12]. According to a recent market report, the global gas sensor market is expected to reach a value of 2.32 billion USD by 2018 [13].

In gas sensors, the active element that is sensitive to the target gas molecules is the key part and thus receives intensive attention [14–18]. For example, after realizing the resistance of some semiconductor materials can be dramatically changed by the presence of gas molecules, many works have been directed to investigate them in gas sensors [19–21]. The conductance of a semiconductor is determined by the carrier concentration and carrier mobility [22]. Gas adsorption should affect one or both parameters to realize gas sensing. Due to the presence of oxygen vacancies some wide bandgap metal oxides, such as ZnO and SnO_2_, are semiconducting and exhibit n-type behavior [3,14,15,21]. In these materials, oxygen vacancies are shallow states and act as n-type donors. The bonding electrons on the adjacent cations can be easily removed and donated to the conduction band. The free electron concentration is high caused by oxygen vacancies. Atoms and molecules prefer to bind at the oxide surface at the oxygen vacancy sites. Some molecules such as oxygen are charge acceptors on the oxide surface. Thus the free electrons in the conduction band can be captured and immobilized by oxygen chemo-adsorption in air. This process lowers the conductance of the oxide. As a result, there is a balance between the free electrons in the oxide body and the captured electrons on the surface. This balance can be modulated by further exposure to oxidizing or reducing gases. The resistance of the oxides can thus be controlled by the adsorption and desorption of gas molecules. The change can be recorded for gas sensing. Usually, in inorganic semiconductors, the modulation of carrier concentration is more important; in contrast, in organic semiconductors, the modulation of carrier mobility is more important.

In resistive-type gas sensors, the main signal comes from the chemistry processes happening on the surface of the sensing element, therefore, active materials with high surface-to-volume ratio are preferred [1,23,24]. Molecular single-walled carbon nanotubes, as a kind of material with extremely high surface area-to-volume ratio, have been found with high oxygen sensitivity [25–27]. Various thick metal-oxide films composed of multi-crystalline grains with very small dimensions have been used in commercialized gas sensors for many years. In 1991, Yamazoe demonstrated that a reduction in the size of SnO_2_ grains would significantly increase the sensor's performance [2]. Nanosized grains of metal oxides are almost depleted of carriers compared to microsized grains in ambient air; hence, when exposed to target gases, much greater modulation of the conductance can be realized. There are two reasons. First, in nanosized grain films, the base carrier concentration is much lower due to the depletion of electrons. Second, more electrons are activated from the captured states to the conduction band due to the much higher surface area-to-volume ratio. Therefore, fabrication of nanomaterials with small dimensions is crucial for gas sensors with high sensitivity.

One-dimensional semiconductor nanostructures, which can be conveniently synthesized by vapor transport and hydrothermal methods or fabricated by lithography and nanoimprint methods, have been intensively investigated due to their unique electronic, optical and mechanical properties [28–40]. The surface area-to-volume ratio of a nanowire could be roughly estimated to be 2/*r*, where *r* is the nanowire diameter. With decreasing diameter, this ratio increases rapidly. In addition, nanowires have much higher aspect ratio compared to grains and thus carriers could transport through drift along the length direction. A lower resistance could thus be attainable. In recent years, many investigations have been made in nanowire-based gas sensors [41–45]. Due to their unique properties, gas sensors with a combination of high sensitivity and low resistance, which is of considerable interest for the practical application, could be realized from semiconducting nanowires [30–32]. In addition, nanowires have promising application in miniaturized gas sensors.

According to the working mechanisms and device structures, semiconducting nanowire gas sensors can be mainly categorized into conductometric and field-effect transistor (FET) types. Conductometic sensors are based on resistance changes caused by the exposure of the sensing elements to target gases. A local heater is usually required to raise the temperature of the sensing material to improve its reactivity. This type of devices is a natural evolution of Taguchi-type metal-oxide gas sensors by replacing the sensing components from metal-oxide grain films to nanowire films. In contrast, FET gas sensors are based on changes in the FET parameters due to the exposure of the sensing channels to target gases. Apart from the current variation, changes in other FET parameters, such as threshold voltage and sub-threshold swing, could also be used for identifying the sensing processes; this is different from the conductometric gas sensors where only the resistance changes are used to realize gas sensing. The FET gas sensors have interesting features include: (1) they are able to work at room temperature (the conductometric sensors are usually working at 200–400 °C); (2) they enable the application of a variety of sensing materials thus enhance the chance to generate a sensing surface for sensitive and selective sensing; and (3) due to the very small dimensions and compatible with micro-fabrication technology, they can be made into sensor arrays for more powerful sensing capability [46]. Several comprehensive reviews are already available about gas sensors based on semiconductor nanowires, especially the conductometric-type gas sensors [41–45].

**Figure 1.**
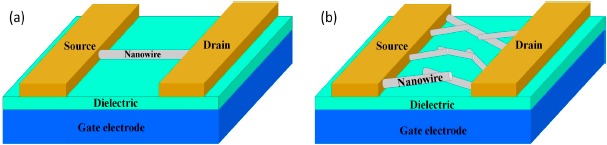
Schematic drawing of nanowire FET gas sensors. (**a**) Individual nanowire as the channel. (**b**) Nanowire network film as the channel.

In this review, we will focus mainly on gas sensors which are based on semiconductor nanowire FETs. As schematically shown in Figure 1, a third gate electrode is used to tune the carrier concentration of the nanowires to realize better sensing performance. Individual nanowires or nanowire network films are usually used as the active detecting elements. The FET parameters can be modulated by the presence of the target gases and their change relates closely to the type and concentration of the gas molecules. In addition, with the help of micro-fabrication techniques, nanowire FET gas sensors can be made very small to be integrated into smart systems. In Section 2, the fabrication of gas sensors and the gas sensing mechanism based on semiconducting nanowire FETs are briefly discussed. In Section 3, recent progress on the research of these gas sensors is summarized. Finally, in the last section, we conclude this review with an outlook of the challenges for future investigation and application. It is worth noting that the term “FET gas sensor” may also refers to metal-oxide-semiconductor FET (MOSFET) gas sensors based on Si or SiC channels. These sensors work on a different sensing mechanism of catalytic interaction between metal gate and gas molecules. A change on the channel current can be measured to give out sensing signals. This is different from nanowire FET gas sensors where the sensing is due to the interaction between the channel and gas molecules. Interested readers may find several excellent reviews on that topic [47,48].

## 2. Fabrication and Mechanism

### 2.1. Structure and Fabrication of Nanowire FET Gas Sensors

Gas sensors based on nanowire FETs usually have the structures depicted in Figure 1. Note that MOSFET sensors work on the principle that molecules entering into the sensor area will be charged positively or negatively, which have a direct effect on the electric field. Introducing each additional charged particle will affect directly the transistor in a unique way, producing a change in the MOSFET signal that can then be interpreted by pattern recognition. Therefore, unlike the top-gate MOSFET gas sensors [47,48], nanowire FET gas sensors usually use an bottom-gate structure. With such a structure, the channel is accessible by the target molecules to serve as the sensing components. Usually, individual nanowires or nanowire network films work as the sensing elements. The fabrication process of these devices includes the synthesis of nanowires and the construction of FET devices.

To synthesize nanowires, there are mainly two approaches: top-down and bottom-up. The top-down approach obtains nanowires from thin film in a controlled way, while the bottom-up approach obtains nanowires from atomic or molecular precursors. Si nanowire is a typical material that can be fabricated by the top-down approach. In addition, there are also some chemical top-down methods [49]. However, except for Si, other semiconductor nanowires such as metal oxides are difficult to obtain by the top-down approach. These nanowires are mainly fabricated by several bottom-up methods, such as vapor phase or liquid phase growth methods. Chemical vapor deposition [50] and vapor transport growth [51] are two main vapor phase methods, while template-assisted deposition [52] and hydrothermal synthesis [53] are typical liquid phase methods. Comprehensive reviews on these topics can be found in literatures [54–56].

The fabrication of nanowire FET gas sensors depends on the way that the nanowires are obtained. The reason for this is that if it is necessary to transfer the nanowires from one substrate to another. In the case of top-down approach, such as using lithographically defined Si nanowires, nanowire fabrication is compatible with the subsequent device making processes. In contrast, if the nanowires are produced by the bottom-up approach, the nanowires have to be transferred onto the final device substrate. This is a difficult process and still needs to be improved. The simplest way is by dispersing the nanowires into a liquid and then dropping the suspending solution on the substrate. This approach gives nanowires lying on the substrate without any control and order. Other methods, such as dielectrophoretic alignment, have been developed to improve the nanowire transferring process [57–60]. The electrodes are usually fabricated by lithography followed by metal deposition. Compared to photolithography, electron-beam lithography can achieve pattern with higher precision [61]. In addition, focused-ion-beam/electron-beam (FIB/EB) direct depositions are also applied occasionally [62]. FIB/EB system uses ion or electron beam to induce decomposition of metal organic precursors and deposit metals in a predefined shape.

Besides above procedures, decoration of nanowires with metal catalyst is frequently required. This can be done either directly after the nanowire growth or when they are transferred to the final substrate. The former case puts less limitation on the preparation condition. Both chemical and physical methods are applicable. The latter case prefers to physical methods, e.g., evaporation or sputtering, to ensure the intactness of the final devices.

### 2.2. Mechanism of Nanowire FET Gas Sensors

Nanowire FET gas sensors work on the principle that adsorption or desorption of gas molecules on the surface of the nanowire will modify the FET parameters such as channel conductance [41–45]. Such a modification is usually realized through tuning the carrier concentration or mobility and depends on the materials used. It is known that the conductance of a semiconductor relates to the concentration and mobility of the free carriers. In a perfect crystal, such as Si and compound semiconductors, free carriers are delocalized. The crystal symmetry is usually disrupted by lattice vibrations, which limits the carrier mobility. In these materials, carriers transport through drift and the conductance is mainly modulated by carrier concentration. However, in an organic crystal, carriers are localized due to defects, disorder or self-localization, here hopping transport is the main conduction mechanism, the tune of the carrier mobility by active gases is much more important for effective sensing.

**Figure 2.**
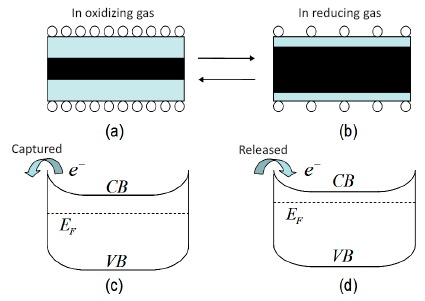
(**a**) In oxidizing gas, some of the free electrons are immobilized on the nanowire surface. (**b**) In reducing gas, some of the trapped electrons are released into the conduction band. (**c**) and (**d**) are schematic energy band diagrams of (a) and (b), respectively.

In the case of metal oxides, the adjustment of the carrier concentration by gas molecules is the main mechanism for gas sensing. Figure 2 shows an example of an n-type oxide nanowire in oxidizing and reducing gases. In an oxidizing gas environment, some of the free electrons in the conduction band are captured on the surface and the free carrier concentration decreases. In contrast, in a reducing gas environment, due to the reaction on the surface, some of the captured electrons are released into the conduction band as free carriers. Therefore, the conductance of oxide nanowires can be tuned by the surface chemical process. In a FET gas sensor, this process can be further tuned by the gate voltage.

Compared with normal conductometric gas sensors, the free carrier concentration in nanowire FET gas sensors can be tuned to enhance the sensing ability. Firstly, the transfer characteristics are dependent on the type of gas for selectivity. In addition, the sensitivity can be tuned by setting the gate voltage to a level that maximum conductance change appears and changes in other FET parameters, including threshold voltage and sub-threshold swing *etc.*, could also be used for identifying the sensing process.

### 2.3. Sensing Performance Advantage

There are several aspects that are important for gas sensors, including gas sensitivity and selectivity, response time, stability *etc.* Semiconductor nanowires are unique system to have good performance on these parameters. At first, the surface of the nanowires is fully exposed to the gas environment. Due to the very large surface area-to-volume ratio, a high sensitivity can be expected. By tuning the base conductance to a low level, very high sensitivity has been reported [63,64]. In addition, metal nanoparticles can be easily decorated with a high coverage ratio on the surface of a nanowire for gas selectivity. With the use of gate voltage as well as heating, light irradiation *etc.*, the response time of the fully-exposed nanowire FET sensors can be accelerated.

## 3. FET Gas Sensors Based on Semiconductor Nanowires

Many kinds of semiconductor nanowires, especially metal oxides of SnO_2_, ZnO and In_2_O_3_, have been intensively investigated in FET gas sensors. In this section, the discussions are divided into five parts, according to the type of materials that are FET gas sensors based on SnO_2_, ZnO, In_2_O_3_ and other oxides, Si, and organic nanowires, respectively.

### 3.1. FET Gas Sensors Based on SnO_2_ Nanowires

SnO_2_-based gas sensors are the predominant solid state sensors used nowadays [3,15]. In 1962, Taguchi applied for a Japanese patent on porous SnO_2_ ceramic material based gas sensors, and this is the very beginning of SnO_2_ gas sensors [65]. After numerous efforts, SnO_2_-based gas sensors were put onto the market in 1968. It has been reported that by decreasing the diameter of the nanocrystals, a much larger change of resistance can be realized [1]. After the successful synthesis of single-crystalline SnO_2_ nanowires, it became possible to investigate this new material in FET gas sensors with the help of micro-fabrication technology. Many works on FET gas sensing has been carried out with SnO_2_ nanowires [55,66–72]. Three particularly impressive works [70–72] on improving the sensitivity and selectivity are discussed below.

**Figure 3.**
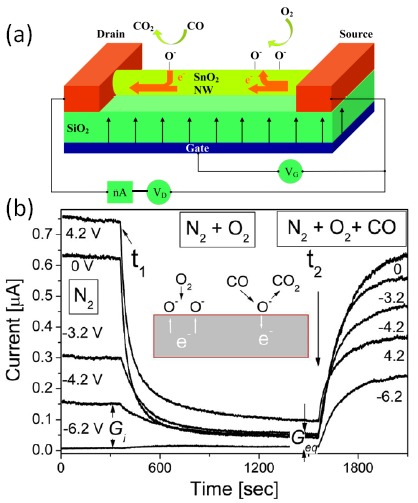
(**a**) A schematic illustration of the nanowire FET gas sensor. (**b**) Evolution of the nanowire conductance in three different gas conditions, at time *t_1_*, N_2_ was changed to a mixture of N_2_ + O_2_, and at time *t_2_* to a mixture of N_2_ + O_2_ + CO. The response curves of the device were drastically tuned by gate potentials, especially at the decaying and rising regions. Reprinted from reference [70] with permission from the American Chemical Society.

Moskovits *et al.* reported that in a typical SnO_2_-nanowire FET gas sensor, as shown in Figure 3a, the rate and extent of oxidation and reduction reaction taking place at the surface of a nanowire can be modified by changing the electron concentration in the wire through the gate voltage [70]. This is a characteristic property of nanowire FET gas sensors.

The response of the device to three different gas atmospheres was investigated. As shown in Figure 3b, when the gas only contains N_2_, oxygen desorbs thermally from the surface of the nanowire, creating surface vacancies. The electrons captured by the oxygen adsorption are thermally excited into the conduction band and the current is high. When oxygen is introduced into the gas flow at *t_1_*, the SnO_2_ nanowire surface adsorbs it at the vacancy sites, and the electrons, that are localized in the vacancy and able to be easily activated into the conduction band, are trapped again. They can no longer contribute to conductance and thus the current decreases. At time *t_2_*, CO is introduced into the gas mixture, which reacts with oxygen ions on the surface and creates oxygen vacancies as new donor states. The current across the nanowire channel increases again. It can be seen that the gas response curves can be tuned by the gate voltage, especially at the decaying and rising regions. By changing the number of electrons available for oxygen surface chemistry, the oxidation rate and the extent of oxygen adsorption can be modulated. When the gate voltage is carefully selected, the sensitivity of the device can be extremely high. Their results showed that manipulating the carrier concentration inside a nanowire affects the chemical reactivity of its surface and are very important for nanowire FET gas sensors.

**Figure 4.**
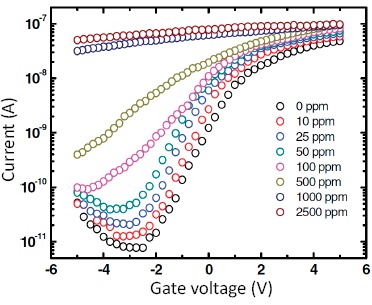
Transfer characteristics of Pd-nanoparticle decorated SnO_2_ nanowire channel under different H_2_ partial pressures. The bias voltage is 0.5 V. Reprinted from reference [71] with permission from John Wiley and Sons.

Decoration of catalytic metal nanoparticles on oxides has been an effective method to improve the activity of oxide surfaces [73–75]. Therefore, the sensing ability of pristine SnO_2_ nanowire can be further enhanced by metal decoration. By using Pd-nanoparticle-coated SnO_2_ nanoiwres as the channel, Moskovits *et al.* obtained an unusually high sensitivity to H_2_ when the device was working in the depletion region [71]. Figure 4 shows the transfer characteristics of the SnO_2_ nanowire channel decorated with Pd nanoparticles under different H_2_ partial pressures at 100 °C.

At a gate voltage of −3 V, the channel current is heavily affected by H_2_. As the H_2_ partial pressure increases from 0 to 2500 ppm, the current increases by nearly five orders of magnitude. This change is extremely high. From the transfer curves, the carrier concentration can be extracted under different conditions. It is found that the response of the device to H_2_ relates directly to the increase of carrier concentration in the nanowire channel. The H atoms can react at a variety of surface sites and potentially diffuse into sub-surface regions of the nanowire to create donors states for increased free carrier density. From the curves in Figure 4, it can be seen that the device exhibits maximal response when the gate voltage is equal to the threshold voltage. This work indicates that in SnO_2_ nanowire FET gas sensors, the combination of the gate and metal catalytic nanoparticle decoration can effectively enhance the sensor's response.

**Figure 5.**
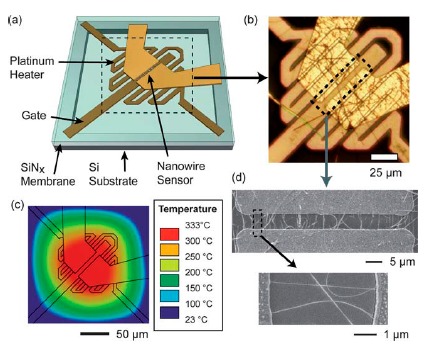
(**a**) The schematic view of the sensor fabricated on a silicon nitride membrane-based micro-hotplate platform. Different components, including Pt heater, gate and nanowire sensor *etc.*, are marked by the arrows. (**b**) An optical image of the device. (**c**) Simulating results at a typical working condition. (**d**) SEM images of the device. Reprinted from reference [72] with permission from the Royal Society of Chemistry.

In addition to gas sensitivity, gas selectivity is another important parameter in gas sensors. Since for a semiconductor channel, various reducing (or oxidizing) gases affect the current across the nanowire channel with the same trend, more controls over the channel are therefore required to realize selectivity. As an interesting example, Dattoli *et al.* combined temperature and gate voltage control to modulate SnO_2_ nanowires [72]. As shown schematically in Figure 5, the device contains a local-heating apparatus which can heat the nanowire to desired temperatures. Together with the gate voltage, the SnO_2_ nanowire sensor can be set to different working conditions to realize selectivity. During the test, the temperature and gating conditions were modulated in order to induce variations to the sensor's response behavior to analyte. By treating the results as identifying fingerprint, analyte identification is achieved using a statistical pattern recognition procedure, linear discriminant analysis. Obviously, this interesting method can be applied to other nanowire systems and are very promising.

From SnO_2_ nanowire FET gas sensors, it can be seen that the gate voltage can tune the response to gases effectively to realize better sensing performance; this is clearly different to the normal two-terminal conductometric gas sensors where the base condition of the channel is fixed. In addition, extremely high sensitivity can be obtained by combining the gate voltage and the catalytic metal decoration. Moreover, gas selectivity can be realized by using both the gate and temperature controls over the nanowire channel. These results indicate that in nanowire FET gas sensors the active channel can be controlled by the gate voltage, catalytic metal nanoparticle decoration and temperature and increased functionality can be realized.

### 3.2. FET Gas Sensors Based on ZnO Nanowires

Like SnO_2_, the resistance of ZnO is also very sensitive to the gas environment and ZnO has been intensively investigated in conventional multi-crystalline film gas sensors [76–79]. Several works on ZnO nanowire FET gas sensors have been reported [23,80–85], although most of them are similar to SnO_2_ nanowires. Here two special works are discussed. One relates to light illumination and another relates to the refresh of the system.

**Figure 6.**
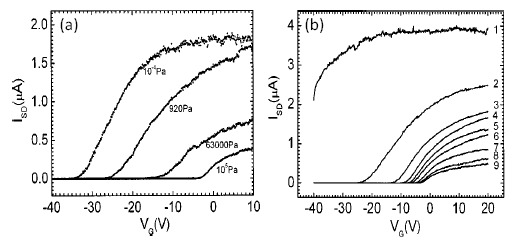
(**a**) Channel current *vs.* gate voltage under different oxygen pressures. (**b**) Curve 1 was obtained under ultraviolet illumination with an oxygen pressure of 10^5^ Pa. Curve 2–9 were obtained 1, 3, 5, 6, 9, 14, 20, 26 min after turning of the ultraviolet light, respectively. In these measurements, the bias voltage is 2 V. Reprinted from reference [86] with permission from AIP Publishing.

Using individual ZnO nanowires, Wang *et al.* fabricated FET sensors and investigated the oxygen sensing properties under ultraviolet illumination [86]. As shown in Figure 6a, the channel current is sensitive to the oxygen pressure. The response of the FET sensors to oxygen is characterized by a shift of threshold voltages as well as a change of channel current. The current decreased and the threshold voltage was shifted to the positive direction with increasing oxygen pressure. In addition, with increasing oxygen pressure, the slope in the linear region decreases. This can be ascribed to increased oxygen ion coverage at the nanowire surface at higher oxygen pressure. They also found out that, in addition to gate voltage, ultraviolet light illumination strongly affects the transfer characteristics, as shown in Figure 6b. Upon illumination, the current at an oxygen pressure of 10^5^ Pa increases a lot, as shown by curve 1 in Figure 6b, due to photo-generated carriers as well as light-induced desorption of surface oxygen ions. Curve 2 is measured 1 min after turning of the ultraviolet light. It takes nearly half an hour for the device to recover. The results indicate that above bandgap illumination can severely affect the oxide nanowire channel which can be further used as an additional control method of nanowire FET gas sensors.

**Figure 7.**
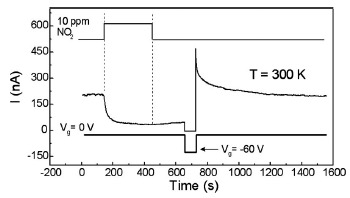
The response of the nanowire to 10 ppm NO_2_. A gate pulse of Minus60 V was used to refresh the surface of the nanowire at room temperature. Reprinted from reference [87] with permission from AIP Publishing.

The quick refresh of the nanowire channel, especially the nanowire surface, is very important for the application. It is difficult to return nanowire chemical sensors to the initial state at low temperature because the thermal energy is normally lower than the activation energy for desorption. However, Lu *et al.* found out that in addition to significantly affecting the gas sensitivity, the gate potential can also be used as an effective method to refresh the nanowire sensors [87], as shown in Figure 7. The pulses of 10 ppm NO_2_ gas and the gate potential are shown by the top and bottom curves, respectively. On exposure to NO_2_ pulse the current across the nanowire decreases gradually. After turning off the NO_2_ gas, the current is still at the low level and increases very slowly. By applying a Minus60 V gate potential, the current increases sharply and then drops gradually to the initial conductance state. The refresh process is markedly accelerated by a large negative gate potential. The interesting result in this work expands our understanding on the gate potential.

### 3.3. FET Gas Sensors Based on In_2_O_3_ and Other Oxide Nanowires

**Figure 8.**
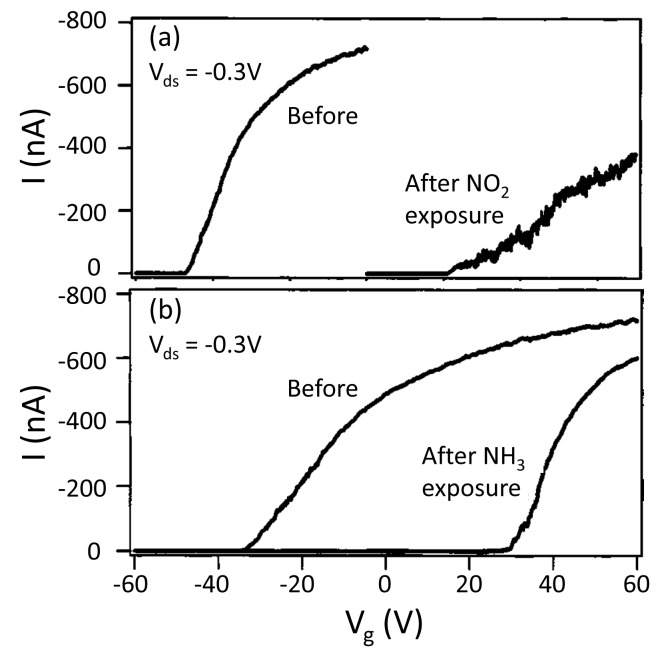
(**a**) Transfer characteristic of the In_2_O_3_ nanowire FET gas sensor recorded before and after NO_2_ exposure. (**b**) Recorded before and after NH_3_ exposure. Reprinted from reference [63] with permission from AIP Publishing.

In_2_O_3_ is another popular material for gas sensors and several works are about In_2_O_3_ nanowire FET gas sensors [63,64,88–90]. Zhou *et al.* reported the first work on individual In_2_O_3_ nanowire transistors as room temperature gas sensors [63]. Upon exposure to 100 ppm NO_2_ in Ar at a bias of Minus0.3V, the shape of the transfer characteristic curve changed a lot and the threshold voltage shifted from Minus48 to 20 V, as shown in Figure 8a. It means that, due to NO_2_, the free carrier concentration of the nanowires decrease dramatically. A similar behavior can be observed for NH_3_ in Ar, as shown in Figure 8b. Note that the transfer characteristics are very different for NO_2_ and NH_3_ gases. By carefully selecting the working gate voltage, sensitivity as high as 10^5^ is reached for NH_3_ detection.

**Figure 9.**
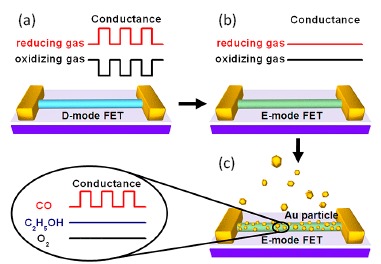
(**a**) Conventional nanowire FET gas sensors that work on the depletion mode. Reducing/oxidizing gas can result in the increase/decrease of conductance. (**b**) If the nanowire channel is deeply depleted, the device has no response to both oxidizing and reducing gases. (**c**) The nanowire surface can be activated by decorating selected catalytic metal nanoparticles for both sensitivity and selectivity. Reprinted from reference [64] with permission from the American Chemical Society.

In order to realized selectivity, Liao *et al.* designed a nanowire transistor that worked at the enhancement mode with the help of decorated nanoparticles [64]. The basic mechanism is very similar to the device shown in Figure 4. As shown in Figure 9a, in a normal n-type oxide nanowire gas sensor, which works on electron transfer between the nanowire and the gas molecules adsorbed on the surface, can response to both reducing and oxidizing molecules, with an increase and decrease of conductance, respectively. If the nanowire channel is tuned to enhancement mode, because the channel is depleted, it cannot detect oxidizing gases. If the channel is depleted much deeper, even reducing gases cannot be detected. Therefore, the device has no response to both oxidizing and reducing gases, as shown in Figure 9b. However, the surface of the nanowire can be activated by decorating selected catalytic metal nanoparticles, such as Au, Pt and Ag, to realize sensitivity and selectivity to CO, H_2_ and ethanol, respectively. An illustration for the Au-decorated case is shown in Figure 9c. An experimental demonstration was performed on gas sensors based on Mg-doped In_2_O_3_ nanowires. In addition to the efficient response to one particular gas, the sensors also exhibited high sensitivity, low power consumption and fast response. The devices can work at room temperature and detect to sub ppm level for reducing gases such as CO.

**Figure 10.**
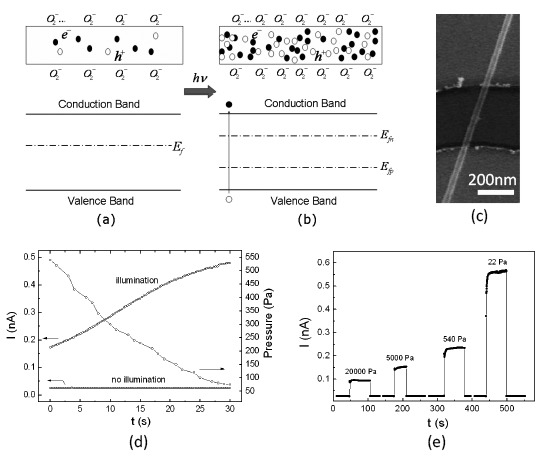
Optically driven oxygen sensing in β-Ga_2_O_3_ nanowires. Schematic and energy-level representations of the nanowires (**a**) without and (**b**) under ultraviolet illumination, E_f_ is the Fermi level, E_fn_ and E_fp_ are the steady-state Fermi level for electrons and holes under the illumination, respectively. (**c**) A SEM image of the nanowire oxygen sensors. (**d**) The current of the device as the oxygen pressure in the chamber changes. The illumination makes the devices become sensitive to oxygen. (**e**) Current-time curves under different oxygen pressures, very fast response and recovery processes can be realized. Reprinted from reference [93] with permission from AIP Publishing.

Several other oxide nanowires have also been reported [91–93]. A special work is mentioned here. As has been discussed, the main purpose of the gate potential is to tune the free carrier density in the nanowire, which is balanced with the captured carriers on the surface. Light illumination can also increase the free carrier concentration in an oxide nanowire and is used to realize optically driven oxygen sensing in low conductance nanowires [93]. The key to fast response is to rapidly tune the carrier density. In a semiconductor, the carrier density can be modulated by above-bandgap light. Under illumination, the oxide nanowire that initially has a high conductance, such as ZnO nanowires, needs a long time to reach equilibrium [86]. Based on such facts, by using low conductance nanowires, an optically driven fast oxygen sensing mechanism was proposed and demonstrated [93]. As shown in Figure 10a, initially, the free carrier density in a nanowire is low; the modulation of its conductance by oxygen will be very small. Under illumination, the current across the nanowire will increase to a value that can reflect the level of the oxygen pressure very quick, as shown in Figure 10b.

When the illumination is off, the photogenerated carriers recombine rapidly and the optically driven oxygen sensing vanishes. By using individual β-Ga_2_O_3_ nanowires as the sensing elements and ultraviolet illumination as a means to excite excess free carriers in the nanowires (just like a field-effect gate done), in the devices shown in Figure 10c, fast response oxygen sensing are realized. As exposed to the 254-nm light, due to the fast electron-hole generation process, the carrier concentration in the nanowire rapidly increased. At this stage, more surface oxygen ions would form. And such an increase of the surface oxygen ion coverage directly correlated to the oxygen pressure. As a result, the current across the nanowire could reflect the level of the oxygen pressure. The sensing results of the devices are shown in Figure 10e. Very fast response and recovery processes were realized.

### 3.4. FET Gas Sensors Based on Si Nanowires

Si nanowires can also be used as channels in FET gas sensors. Due to the presence of an insulating native oxide layer on the surface, Si nanowires work in an analogous way to a FET. The electrostatic potential on the surface of the nanowire controls the free carrier concentration in the channel as an extra gate. By careful design, various kinds of receptors can be functionalized on the surface to detect the target molecule which can be recognized by the receptor for selectivity [94,95]. In most cases, nanowire synthesis and device fabrication are performed on separate substrates, especially for oxide nanowires, which will face the difficulty of controlled transfer of the nanowires. In addition to catalyst directed vapor-liquid-solid growth route, Si nanowires can be made by lithography techniques. This is a very special characteristic of Si nanowires.

**Figure 11.**
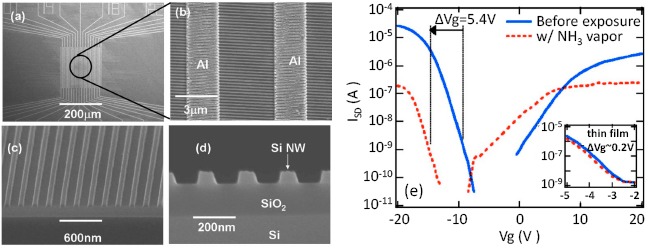
(**a**) Top-down image of one Si nanowire device with interdigitated Al source and drain electrodes. (**b**) Close-up SEM image, the dense, well-arranged Si nanowire channels were connected with two Al electrodes. Cross-sectional SEM images at (**c**) 60° and (**d**) 90° tilt. (**e**) Channel current *vs.* gate voltage curves before and after NH_3_ exposure, a negatively shift of the threshold voltage of about 5.4 V was obtained after NH_3_ exposure; for comparison, the inset shows that the SOI thin film device has much smaller threshold voltage shift. Reprinted from reference [96] with permission from AIP Publishing.

Based on silicon-on-insulator (SOI) wafers and nanoimprint lithography, a top-down approach was used by Talin *et al.* to fabricate Si nanowire FET gas sensors [96]. Large-area, dense p-type nanowire array sensors are shown by the SEM images in Figure 11a–d. The Si nanowires are well defined with diameters of about 76 nm and oxidized with O_2_ plasma treatment. The nanowire channel exhibited similar mobility to the corresponding thin film device, indicating no significant degradation of the Si nanowire body during the dry etching process. The response (defined as the shift of threshold voltage) of the Si nanowire FET sensors to NH_3_ vapor is shown in Figure 11e. Due to the reducing character of NH_3_, a negative shift of the threshold voltage by about 5.4 V was observed. In contrast, a thin film device shows a much smaller threshold voltage shift, as shown in the inset of Figure 11e. This top-down approach could be very promising in the investigation of Si nanowire FET gas sensors.

**Figure 12.**
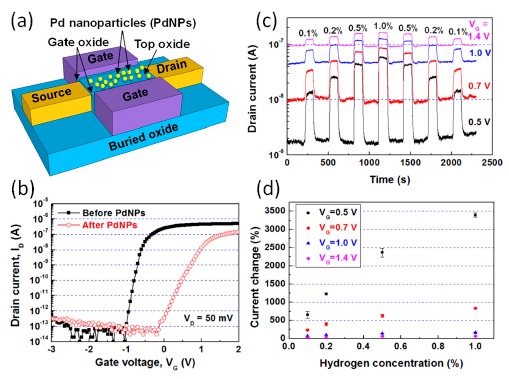
(**a**) Schematic illustration of the Pd-decorated Si nanowire FET gas sensor with local side gates. (**b**) Transfer characteristics of the pristine and Pd-decorated Si nanowire FET gas sensors. (**c**) Real time measurement of the current across the channel in response to H_2_ pulses at different concentrations under four gate voltages. (**d**) Current change *versus* H_2_ concentration, the current change is defined as (I_H2_-I_air_)/I_air_*100%, where I_air_ and I_H2_ are the current in air and H_2_, respectively. Reprinted from reference [98] with permission from AIP Publishing.

Using the fabrication processes similar to the FinFET [97], an n-type Si nanowire FET with two side-gates was fabricated by Park *et al.* [98] as shown in Figure 12. The nanowires' sensing/top surface was oxidized by annealing and passivated with H bonds. The device has a channel width of 100 nm and a gate length of 1 μm. The side-gates were insulated from the nanowire channel by thermally grown SiO_2_ of about 5 nm thick. To enhance the H_2_ selectivity and sensitivity, the nanowire top surface was further deposited with a ∼1 nm thin Pd layer. During the operation, two side gates were applied with the same voltage.

The transfer curve in Figure 12b shows an increase of threshold voltage and a decrease of channel current, indicating the enhanced extraction of electrons from the channel after Pd decoration. As can be seen in Figure 12c–d, the Pd decorated sensor shows an evident gate voltage dependent H_2_ sensing. Higher response is found when the device is working in the sub-threshold region (V_G_ = 0.5 V). In contrast, pristine Si nanowire device exhibits no response to H_2_ up to 1%. Such a comparison indicates the strong activation effect of Pd in the decorated device. H_2_ will dissociate on the surface of Pd, producing H atoms, which then diffuse into the Pd nanoparticles and generate hydrogen-induced dipole layers at the interface. The positive dipole layers eventually cause the increase of conductance. The gate voltage dependence response once again evidences that high response is more obtainable at a low electron concentration state than a high electron concentration state, as demonstrated in Figure 4 and Figure 9.

### 3.5. FET Gas Sensors Based on Organic Nanowires

**Figure 13.**
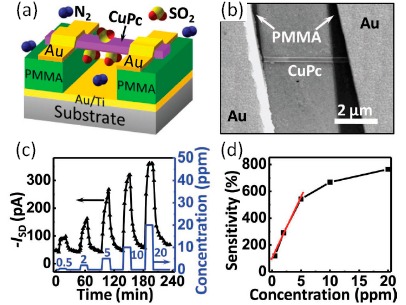
(**a**) Schematic illustration of the CuPc nanowire FET sensors. (**b**) A SEM image of the device, the nanoawire was floated up by PMMA. (**c**) The response of the device as the SO_2_ concentration was changed from 0.5 to 20 ppm. The gate voltage was Minus10 V and the bias Minus15 V. (**d**) The sensitivity of the device. Reprinted from reference [99] with permission from John Wiley and Sons.

In the inorganic nanowires shown above, the modulation of the free carrier concentration by the surface chemical process is the main mechanism for gas sensing. It is different when an organic nanowire is used as the channel, because in organic semiconductor hopping transport dominates. Several works are about organic nanowire FET gas sensors [99–105]. A typical work is discussed here. By using single crystalline CuPc nanowires, room temperature SO_2_ sensing with low detection level down to sub ppm regions was reported by Liu *et al.* [99]. In their system, the CuPc nanowire was floated from the substrate, thus the whole channel was exposed to the gas atmosphere, as shown by the illustration in Figure 13a and the SEM image in Figure 13b.

The device was in pure dry N_2_ at first. On exposure to SO_2_ pulses, as shown in Figure 13c, the current had a rapid response followed by a gradual recovery. The sensitivity was defined by the ratio between current change and the base current. The device exhibited a fascinating sensitivity of 119% to 0.5 ppm SO_2_. In organic materials, the transport refers to hopping of charge carriers between molecules. Carrier mobility is reliant upon the levels of similar energy for the carriers to move on. This process can be affected by a variety of parameters. In this research, the authors ascribed the increase of current upon SO_2_ exposure to the compensation of shallow traps in the CuPc nanowire. Since the mobility of organic conductors can be changed over a very large range, it is possible to realize high sensitivity by tuning the carrier mobility, which is in contrast to inorganic semiconductors where carrier concentration tuning is more crucial.

## 4. Conclusions and Outlook

A lot of works have been reported on semiconducting nanowire FET gas sensors as shown above. Active gas molecules affect the nanowire's conductance by mainly tuning the free carrier density, such as in oxides, or the carrier mobility, such as in organic materials. In contrast to normal nanowire sensors with two-terminal structures, a gate electrode was used to tune the free carrier density. With this method, the balance between the free carrier concentration in the nanowire and the surface chemo-sorption of active molecules was modulated and enhanced sensing performance could be realized. These researches are helpful for better understanding of semiconductor nanowires on the correlation between free carrier concentrations and surface chemistry, which are important for their application in gas sensors. Based on these progresses on nanowire FET gas sensors, it is necessary to highlight two things, the basic tuning mechanism and the fabrication procedure of the device, which are important for future investigation.

**Figure 14.**
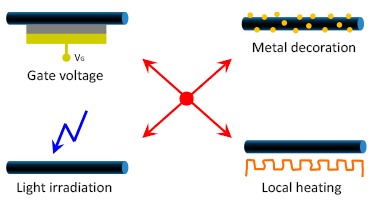
In addition to gate voltage, other methods including metal particle decoration, light irradiation and local heating can be used as effective additional means to modulate the nanowire channel in the FET gas sensors for improved sensing performance, such as sensitivity, selectivity and response time.

Firstly, we comment on the basic tuning mechanisms that have been used. In the nanowire FET gas sensors, the chemical process at the nanowire surface can affect the transfer characteristics. Therefore, by appropriately choosing the gate voltage, the carrier concentration in the nanowire can be tuned and the sensing performance can be improved. In addition to the gate voltage, other methods can also be used as extra controls over the nanowire channel for gas selectivity and sensitivity, response time *etc.* Figure 14 summarizes four methods that are used and discussed in the above section. In addition to the gate voltage, metal nanoparticle decoration, light irradiation and local heating are also efficient tuning methods. Metal decoration and local heating can increase the surface activity of the nanowire, while above-bandgap irradiation can result in a sharp increase of the carrier concentration and a reset of the surface condition.

Secondly, we comment on the fabrication procedure of the devices. Except for the nanowires, all other components of FET gas sensors, such as metal electrodes, local-heating apparatus, metal decoration etc, are compatible with conventional micro-fabrication technology. Therefore, controlled alignment and patterning of the nanowires at a desired position on a large area is very important for controlled fabrication of the nanowire FET gas sensors. For the preparation of the nanowires, there are two approaches, top-down and bottom-up schemes. The top-down approach is preferred because the nanowires can be well-defined in a controllable way. Si nanowires can be obtained by the top-down method; however, the processes still need to be refined for much smaller diameter and improved sensing performance. Various kinds of oxide nanowires are usually synthesized by the bottom-up method. However, high-speed, large-area patterning of nanowires with good control on the orientations and dimensions are still a significant challenge.
